# A qualitative study on health care providers’ experiences of providing comprehensive abortion care in Cox’s Bazar, Bangladesh

**DOI:** 10.1186/s13031-021-00338-9

**Published:** 2021-01-13

**Authors:** Maria Persson, Elin C. Larsson, Noor Pappu Islam, Kristina Gemzell-Danielsson, Marie Klingberg-Allvin

**Affiliations:** 1 Department of Women’s and Children’s Health, Karolinska Institutet, Widerströmska Huset, Floor 8, Tomtebodavägen 18A, SE-171 77 Stockholm, Sweden; 2 Department of Women’s and Children’s Health, Uppsala University (Akademiska Sjukhuset), SE-751 85 Uppsala, Sweden; 3 Dalarna University School of Education, Health and Social Studies, Högskolegatan 2, SE-791 88 Falun, Sweden; 4 WHO Centre, Karolinska University Hospital, WHO Centre, QB:84, Karolinskavägen 37A, SE-171 76 Stockholm, Sweden

**Keywords:** Comprehensive abortion care, Menstrual regulation, Humanitarian setting, Health care providers, Humanitarian actors, Policy, Bangladesh, Rohingya

## Abstract

**Background:**

Humanitarian settings are characterised by limited access to comprehensive abortion care. At the same time, humanitarian settings can increase the vulnerability of women and girls to unintended pregnancies and unsafe abortions. Humanitarian actors and health care providers can play important roles in ensuring the availability and accessibility of abortion-related care. This study explores health care providers’ perceptions and experiences of providing comprehensive abortion care in a humanitarian setting in Cox’s Bazar, Bangladesh and identifies barriers and facilitators in service provision.

**Method:**

In-depth interviews (*n* = 24) were conducted with health care providers (*n* = 19) providing comprehensive abortion care to Rohingya refugee women and with key informants (*n* = 5), who were employed by an organisation involved in the humanitarian response. Data were analysed using an inductive content analysis approach.

**Results:**

The national menstrual regulation policy provided a favourable legal environment and facilitated the provision of comprehensive abortion care, while the Mexico City policy created organisational barriers since it made organisations unable or unwilling to provide the full comprehensive abortion care package. Supplies were available, but a lack of space created a barrier to service provision. Although training from organisations had made the health care providers confident and competent and had facilitated the provision of services, their knowledge of the national abortion law and menstrual regulation policy was limited and created a barrier to comprehensive abortion services. Even though the health care providers were willing to provide comprehensive abortion care and had acquired skills and applied strategies to communicate with and provide care to Rohingya women, their personal beliefs and their perceptions of Rohingya women influenced their provision of care.

**Conclusion:**

The availability and accessibility of comprehensive abortion care was limited by unfavourable abortion policies, a lack of privacy, a lack of knowledge of abortion laws and policies, health care providers’ personal beliefs and a lack of cultural safety. To ensure the accessibility and availability of quality services, a comprehensive approach to sexual and reproductive health and rights is needed. Organisations must ensure that health care providers have knowledge of abortion policies and the ability to provide quality care that is woman-centred and non-judgmental.

**Supplementary Information:**

The online version contains supplementary material available at 10.1186/s13031-021-00338-9.

## Background

Humanitarian settings are often characterised by limited access to comprehensive sexual and reproductive health (SRH) services, including safe abortion services [1, 2]. Hence, humanitarian settings increase the vulnerability of women and girls to unsafe abortions due to reduced access to SRH services and supplies as a consequence of the collapse, weakening or disruption of the health system and the provision of health care [1, 3, 4]. Moreover, during humanitarian crises, patterns of discrimination and sexual violence against women and girls are aggravated, resulting in an increased risk of unintended pregnancies and unsafe abortions [3, 4]. Unsafe abortion is one of the main causes of maternal mortality and morbidity, accounting for 7.9% (4.7–13.2%) of all maternal deaths globally, and it is associated with severe complications that can lead to temporary or permanent disability [5]. International recommendations on sexual, reproductive, health and rights (SRHR) in humanitarian settings highlight the importance of incorporating a comprehensive approach to SRHR, including comprehensive abortion care (CAC) services, which comprise safe abortion care, post-abortion care (PAC) and the provision of contraceptives and contraceptive counselling, to reduce the risk of maternal mortality and morbidity as a consequence of unintended pregnancies and unsafe abortions [2].

Abortion is one of the safest medical procedures when performed according to recommended medical guidelines [2]. Abortion safety is defined along a continuum: safe abortions are those performed by a trained person using a method recommended by the World Health Organisation (WHO); less safe abortions are those where only one of the two criteria for safe abortions are met (i.e. either a trained provider is present or a recommended method is used); and least safe abortions are those where none of the criteria for safe abortions are met [6]. Although there has been overall growth in the capacity for reproductive health care in humanitarian settings as well as in the availability of reproductive health services, the provision of CAC services remains limited [1, 7, 8]. At an institutional level, the limited access to safe abortions is due to the perceptions that ‘there is no need’, ‘abortion is too complicated to provide in crises’, ‘abortion is illegal’ and ‘donors do not fund abortion services’ [9]. In addition, a lack of training of health care providers (HCPs), a lack of knowledge about the legal status of abortion in the specific context and humanitarian actors’ and HCPs’ stigmas about and negative attitudes toward abortion constitute additional barriers to safe abortion services in humanitarian settings [1, 10, 11]. However, Gasseer et al. [3] highlight the positive effects that reproductive HCPs in humanitarian settings can have on women’s health outcomes. Research in low- and middle-income countries (LMIC) has also shown that HCPs play an important role in the provision of comprehensive abortion care and that their personal perception of abortion and attitudes toward women seeking abortion-related care can act as both a facilitator and a barrier in service provision [12, 13].

### The humanitarian setting in Cox’s Bazar, Bangladesh

The world’s most densely populated refugee settlement is located in Cox’s Bazar district in Bangladesh and is home to over 900,000 Rohingya refugees [14]. Rohingyas are a Muslim ethnic minority, predominantly from the western Rakhine State of Myanmar, which is close to the Bangladeshi border. Since August 2017, more than 745,000 Rohingya refugees have fled Myanmar due to ongoing persecution and escalating violence, joining the estimated 200,000 Rohingyas already residing there [14]. The congestion in the settlement has led to poor living conditions and affected the health and well-being of the Rohingya population [14]. Rohingya women and girls currently living in the refugee settlement in Cox’s Bazar have been exposed to sexual violence, both before and during their flight. The United Nation’s (UN) independent fact-finding mission in Myanmar reports large-scale incidences of rape and other forms of sexual violence by the Myanmar military, targeting Rohingya women and girls [15]. Furthermore, following displacement, Rohingya women and girls continue to face different forms of sexual- and gender-based violence [14], which might further increase the risk of unintended pregnancies and unsafe abortions.

In Bangladesh, abortions are restricted unless necessary to save the mother’s life [16]. However, menstrual regulation (MR), a procedure for regulating the menstrual cycle to ensure a non-pregnancy, is permitted up to weeks 10–12 following a woman’s last menstrual period (LMP) [17, 18]. Trained paramedics, nurses, midwives and Family Welfare Visitors (FWVs) can provide MR up to week 10 and doctors up to week 12 following a woman’s LMP. In contexts where abortion is restricted due to social, cultural or religious norms, MR may be considered more acceptable [19]. MR, PAC and family planning (FP) services are provided throughout Bangladesh and to the Rohingya population in the humanitarian setting in Cox’s Bazar [20]. In Bangladesh, MR is performed either through manual vacuum aspiration (MVA) or medication, using a combination of mifepristone and misoprostol [18], both of which the WHO recommends as safe abortion methods [21, 22]. CAC services in the form of MR, PAC and FP were established early in the crisis and later scaled up to 29 primary health centres (PHC) as part of the minimum initial services package (MISP), a set of minimum standards for SRH response in humanitarian crises [11, 20]. However, the settlement is characterised by infrastructural challenges, such as poor roads and a lack of transportation, limiting movement. Further, there is limited access to essential information, and social and cultural norms restrict Rohingya women’s and girls’ movement outside the shelter [14], which affects their access to MR, PAC and FP services.

In Bangladesh, research into MR, PAC and FP has mainly focused on non-humanitarian settings [17, 23, 24]. The research has found that women and girls face barriers to accessing MR, PAC and FP services. Social norms and stigmas limit their access [23], and HCPs have been found to impose preconditions not stated in the national MR guidelines, such as having a husband’s permission [17]. Further, a lack of trained providers and a lack of capacity of health care facilities have been found to limit the access to and availability of MR and PAC [17]. Additionally, HCPs’ personal values have led to women being denied services and affected the quality of care provided [17, 23, 24]. Research into CAC in humanitarian settings is limited in general, and few studies have explored HCPs’ experience of providing CAC in such settings. As humanitarian settings differ from non-humanitarian settings, ongoing research in humanitarian settings is needed to contribute to evidence informing policy, donors and humanitarian actors. Thus, this study explores HCPs’ experiences and perceptions of providing CAC as well as the barriers and facilitators in service provision in the humanitarian setting in Cox’s Bazar, Bangladesh.

## Method

### Study design and setting

This was an exploratory qualitative study involving in-depth interviews (IDIs) with HCPs and key informants (KIs).

The study was conducted inside the refugee settlement in Cox’s Bazar district, Bangladesh. The refugee settlement is divided into 34 camps, which are located in two Upazilas (sub-districts) of Cox’s Bazar, Teknaf and Ukhiya, where most Rohingya refugees have settled [14]. The Government of Bangladesh (GoB) leads the humanitarian response in collaboration with the International Organisation of Migration (IOM) and the Office of the United Nations High Commissioner for Refugees (UNHCR) [14]. In 2019, health care services were provided at 134 health posts, 29 primary health centres, field hospitals and specialised centres within or close to the camps, either managed by the GoB or by a non-governmental organisation (NGO), and at district and sub-district facilities outside the camps [25]. The United Nations Population Fund (UNFPA) leads the coordination of the SRH working group and the SRH’s response in Cox’s Bazar, which in 2019 involved over 50 actors [25]. To access the refugee camps, we needed permission from the Refugee Relief and Repatriation Commissioner (RRRC), a government agency working with the humanitarian response. Authentication from an implementing organisation was also needed and was provided by Ipas, an international NGO involved in the humanitarian response and the provision of MR, PAC and FP in Cox’s Bazar.

### Study participants

The participants were identified through purposeful sampling, meaning that they fit the pre-defined parameters of the study (Table 1). In total, 24 IDIs were conducted, including five with key informants and 19 with HCPs (Table 2). Ipas, the organisation that assisted in gaining access to the refugee camps, also assisted in identifying HCPs who were eligible for the study and in scheduling the interviews. The study objective and inclusion criteria were discussed with Ipas during a preparation meeting. The key informants (*n* = 5) were representatives from organisations supporting the provision of CAC and were identified through the researchers and collaborating organisations.

**Table 1:** Inclusion criteria

Inclusion criteria for HCPs	Inclusion criteria for key informants
• Employed by an organisation providing or supporting the provision of MR, PAC and FP in the humanitarian setting in Cox’s Bazar. • Being a paramedic or doctor. • Working with, providing or supporting the provision of MR, PAC and FP services in the humanitarian setting in Cox’s Bazar.	• Being a senior manager or senior member of staff or a coordinator or programme manager working either directly or indirectly with the humanitarian response in Cox’s Bazar. • Employed at an organisation supporting the provision of MR, PAC and FP as part of the humanitarian response in Cox’s Bazar.

**Table 2:** Study participants’ characteristics

Health care providers	***N = 19***
Occupation	
Doctor	3
Paramedic	16
Work Experience (total)	
0–5 years	9
5–10 years	7
10 years and above	3
Work Experience Humanitarian Setting	
0–1 year	8
1–2 years	10
2–3 years	1
Type of Facility	
NGO-run facility	12
Govt-run facility	5
NGO Hospital	2
**Key informants**	***N = 5***
Gender	
Female	1
Male	4
Occupation	
Sr Manager/Sr Staff	3
Program Manager/Coordinator	2
Occupational setting	
Cox’s Bazar	2
National	1
International	2

In the study setting, MR is mainly provided by paramedics. A paramedic is a medical HCP who has completed a paramedic course, the duration of which varies from 20 months to 3 years and covers basic primary health care, which may include MR, PAC and FP. Paramedics have been deployed at the health care facilities in the camps in Cox’s Bazar for the purpose of providing MR (up to 10 weeks), PAC and FP services. Therefore, a majority of the HCPs who were interviewed were paramedics employed by an NGO, the Association for the Prevention of Septic Abortion, Bangladesh (BAPSA). Doctors were included, as the administration of implants and MR up to week 12 is limited to doctors. The doctors providing and/or supporting the provision of MR, PAC and FP were employed by IOM. The scarcity of doctors providing or supporting the provision of MR, PAC and FP in Cox’s Bazar and their high workload resulted in only three interviews. The paramedics and doctors providing MR, PAC and FP are women in this setting, and thus all interviewed HCPs were women. Some male government doctors are involved in the administration of implants but not in MR and PAC, and thus they were not included in the study.

### Data collection

The data were collected in December 2018 and March 2019. IDIs are well suited for the purpose of this study, as they allow the participants to share their experiences, views and understandings. The interviews with key informants not based in Cox’s Bazar were carried out through Skype (*n* = 3) in December 2018, and those with key informants based in Cox’s Bazar were carried out face-to-face (*n* = 2) in March 2019 in Cox’s Bazar. The Skype and face-to-face interviews were conducted in a private space and treated the same; if consent was given, the interviews were recorded and later transcribed verbatim. All but one interview with key informants were recorded (the reason for this non-recorded interview was that safe abortions is a sensitive topic). The interviews with HCPs (*n* = 19) were carried out face-to-face in March 2019 in the refugee camps in the humanitarian setting in Cox’s Bazar. All but one of the interviews with HCPs were recorded and transcribed verbatim (the reason was not given). In the two cases in which the interviews were not recorded, notes were taken during the interviews.

The average length of the interviews was 50 min (17–120 min). All interviews with key informants and HCPs were conducted in English by one of the female authors (MP). The author conducting the interviews has a background in social science and global health and is trained in qualitative interviewing techniques. Sixteen of the nineteen IDIs with HCPs were conducted together with a female interpreter. The author conducting the interviews held a preparation meeting with the interpreter to explain the data collection process, the study objective and the roles of the interviewer and the interpreter. Further, after the first day of data collection a longer de-briefing session was held to go through the recordings and discuss the interviews. Daily de-briefing sessions were held throughout the data collection period. As all the HCPs interviewed were women, both the author conducting the interviews (MP) and the interpreter were women to ensure comfort during the interviews; neither were employed by an organisation working in Cox’s Bazar. The interpreter translated the questions orally from English to Bengali and the participants’ responses from Bengali to English. To ensure quality translation of the participants’ answers, two English and Bengali speakers transcribed and translated the full interviews verbatim.

The interviews with HCPs were conducted in or close to a health care facility in the refugee camps. Not all HCPs worked at the specific facility where the interviews took place. Due to the infrastructure and facility set-up, with limited rooms and bamboo or tin walls, it was not always possible to ensure complete privacy. For the researchers, it was important to ensure that the participants felt comfortable even if complete privacy could not be maintained at all times and that they understood that their participation was voluntary.

All interviews were conducted based on a pre-defined topic guide (see Additional file 1 for HCPs and Additional file 2 for key informants). Before participation, all respondents in face-to-face interviews provided verbal and written consent, and Skype respondents provided verbal consent. All participants received comprehensive oral and written information about the study’s objectives and methods. They were assured that confidentiality would be maintained, that they had the right to discontinue the interview at any point and that partaking in the study would not negatively affect their current or future work. Finally, they were given information about the risks and potential benefits of the study.

### Data analysis

A content analysis, based on an inductive approach, was conducted to analyse the data [26]. The transcribed interviews were imported into NVivo 12, a qualitative data-analysis software programme, which was used to organise, code and categorise the interview text. The authors read the interview transcripts several times while listening to the recorded interviews to understand the flow of the conversation, including pauses and hesitations. The text was then systematically coded, categorised, further interpreted and analysed. This was not a linear process but required ongoing analysis and returning to the codes, text and context. The data were analysed and interpreted by a team of researchers from Sweden and Bangladesh with multidisciplinary academic backgrounds, including anthropology, social science and medical science. The interpreter was not involved in the data analysis.

## Results

The following three main categories emerged from the data analysis: (1) organisation, collaboration and policies influencing the provision of CAC; (2) the influence of confidence, competence and pride on HCPs’ provision of CAC; and (3) the influence of HCPs’ understanding of Rohingya women’s needs on CAC provision (category system Additional file 3). The categories are presented below and illustrated with quotations. To illustrate which respondent the quotes belong to, each respondent was randomly assigned a number between 1 and 50, which does not correspond to the interview order. The barriers and facilitators in the provision of CAC are summaried in Table 3.

**Table 3:** Barriers and facilitators in the provision of CAC

Category	Barriers	Facilitators
Organisation, collaboration and policies influencing the provision of CAC	• Mexico City Policy • FP policy • Lack of space to provide services • Lack of accessibility and availability of IUD and implant • MR medication sold at local shops	• MR policy • UNFPA’s leadership • Good collaboration among different stakeholders • Supportive work environment • Availability of commodities and equipment
Influence of confidence, competence and pride on HCPs’ provision of CAC	• Lack of knowledge on the abortion law • Varying knowledge on the MR policy	• HCPs confidence • Training of HCPs • Provider–client communication • HCPs taking pride in their work
Influence of HCPs’ understanding of Rohingya women’s needs on CAC provision	• HCPs’ condescending attitudes and preconceived ideas • HCPs’ strategies for increasing acceptance • Requiring husbands’ permission	• HCPs’ strategies for increasing acceptance

### Organisation, collaborations and policies influencing the provision of CAC

The key informants highlighted that the MR policy provides a favourable legal environment in Bangladesh that contributed to facilitating the provision of CAC in the humanitarian setting in Cox’s Bazar. The informants further emphasised UNFPA’s strong leadership in the SRH working group as a facilitating factor in the provision CAC.

I think that there are number of factors [for which CAC can be provided in the Rohingya camps]. I think that a strong leadership from UNFPA, a strong partnership with the Bangladesh ministry of health and with the government and their interest in providing consistent care to the same standards as they are providing Bangladeshi people. (Key informant #31, Sr Manager/Sr Staff).

The collaboration and coordination between organisations working with SRH in Cox’s Bazar was viewed as good by both key informants and HCPs. One paramedic expressed that there was an understanding among the organisations working in the camps: ‘we are working together with a lot of NGOs and we have a good understanding among us such as RTMI, Gonoshasthaya, IRC, Ipas, all these organisations work together’ (#37, 0–5 years of experience). Further, the HCPs reported a supportive work environment at the facility level, which also facilitated the provision of CAC.

Because of the environment here, I can offer all the services. If the clients come, I will definitely provide them the services. I have the instruments, my own place to provide the services […] And the doctors, and everyone is helping me here. (Paramedic #44, more than 10 years of experience).

Both key informants and HCPs stated that MR medications and contraceptives were readily available and that the available supplies and equipment were adequate. However, the key informants and the HCPs reported that MR medications were available in local pharmacies/shops and that buying MR medication from an untrained pharmacist/shop keeper was a reason for incomplete and/or unsafe abortions. As observed by one of the doctors (#14, 0–5 years of experience) ‘It [unsafe abortion] is common [ …] Most common reason is medicine from a local pharmacy’. Further, implants, which can only be administered by doctors in Bangladesh, were accessible only when a doctor was available, which was not on a daily basis. One doctor also reported not having IUDs in stock or the space to administer IUDs.

… implant is not provided here daily. It is not a regular task here […] and we do not have any supply of IUDs. So, IUD is not that popular, also because this facility is very crowded and we only have one bed for delivery, ANC, PNC services and FP services. (Doctor #42, 0–5 years of experience).

The lack of space was described by some of the HCPs as a major challenge and a barrier to providing MR, PAC and FP services both in the NGO- and GoB-managed facilities.

There is no other organisation providing MR and PAC services […] I am not providing the best because, I have told you that we are working in someone else’s hospital, and we do not have a place to sit here, neither can we maintain the privacy. (Paramedic #1, 6–10 years of experience).

The paramedics who were employed by BAPSA were the only service providers who provided first trimester MR services in Cox’s Bazar. Although, other organisations had the capacity to provide MR services, the Mexico City Policy made these organisations unwilling or unable to do so. According to one of the key informants, the Mexico City Policy resulted in CAC services being divided among different organisations; while several organisations/actors were involved in the provision of FP services, MR services were only provided by BAPSA.

..the other NGOs that are providing comprehensive [health care] services in these clinics also have the capacity to provide MR, but given the Mexico City Policy they do not want to provide MR services, but they do not have objections to another provider providing services […] what happens is that there are duplications in the sense that they want to keep the MR outside their purview but want to retain the FP territory. (Key Informant #36, Sr Manager/Sr Staff).

### Influence of confidence, competence and pride on HCPs’ provision of CAC

HCPs felt confident that they provided quality services and that they were adequately trained in MR, PAC and FP. The confidence and quality of services were related to the provision of services without complications, the clients accepting the services provided and the clients coming back for follow-up services, which was described as a result of the HCPs’ counselling skills and their ability to establish trust and rapport.

I am pleased that I am able to provide them [Rohingya women] family planning services and they are listening to my advice now, they understand, recognise and look for me nowadays. The difference is made by us who work here, it is because of our proper counselling. (Paramedic #21, 6–10 years of experience)

The HCPs used different strategies to create trust and establish rapport, such as identifying with the women and trying to soothe those who were scared. Furthermore, the HCPs who spoke a dialect similar to the local Rohingya language used it during counselling.

I can speak their [Rohingya women’s] language well, and I understand every word they say. When I explained everything to her in detail, about the benefits that she will get if she takes the service [IUD], she started to trust me completely even though she was scared at first. (Paramedic #7, 6–10 years of experience)

The HCPs expressed pride in their work and in serving the Rohingya women in Cox’s Bazar. They defined their work as lifesaving and believed that, through their services, they were contributing to a better life for Rohingya women.

… she cried a lot and told me that she is not ready to bear the child […] she was so sacred of the matter being exposed to her neighbours.[…] I feel happy that from a worst-case scenario I helped a girl to live a new life. I love these things about the job, to help and to protect people from danger. (Paramedic #48, 0–5 years of experience)

All but one HCP had received training in MR, PAC and FP from an NGO prior to employment in Cox’s Bazar, which facilitated their provision of CAC. However, knowledge and interpretation of the MR policy differed among HCPs. Not all HCPs knew the gestational age range within which different medical professions were legally allowed to carry out an MR, and when asked about abortion law in Bangladesh, the HCPs either did not know or did not remember the law. Some key informants highlighted the importance of supporting people in understanding the indications for which abortions can be legally provided, and that abortion laws and policies can be interpreted in different ways.

… the first challenge to overcome is to help people to understand that ‘it [abortion] is not illegal’, we do have indications [for which abortion is legal] and to know what those indications are and how we fulfil our duty to women, as health care providers, to provide [abortion services] at every indication that we can. (Key Informant #50, Sr Manager/Sr Staff.

The key informants and a few of the HCPs indicated that there was no difference between abortion and MR, but they used the term MR in Bangladesh. Although the pregnancy was established before providing MR, most HCPs described MR as regulation of the menstrual cycle, which was defined as something different from terminating a pregnancy. Before a certain gestational age (which differed from seven to 12 weeks), the embryo was not considered a child and thus not sinful to abort. As described by one of the doctors (#14, 0–5 years of experience): ‘MR is before the heartbeat. [ …] People see it [MR] as a period regulation thing and not as terminating a pregnancy’.

### Influence of HCPs’ understanding of Rohingya women’s needs on CAC provision

Some of the HCPs perceived the Rohingya community as patriarchal and Rohingya women as religious and conservative, which occasionally led them to describe the women (and their community) in a condescending way.

They [Rohingya] want more children, their husbands’ want more children. He wouldn’t allow these things [family planning]. And their religious mindset. And they are totally illiterate, they do not know about family planning. (Paramedic #7, 6–10 years of experience)

The view of the Rohingya community as patriarchal led HCPs to require husbands’ permission before providing services. Some HCPs perceived this as a norm in the Rohingya community and a way to protect women, and for others it was part of their routine counselling. However, the HCPs also reported providing services without a husband’s permission and caring for women who wanted to keep their FP or MR a secret from their husbands and families. Another reason that some HCPs considered a husband’s permission was fear of repercussions, which was also noted by a key informant.

… some men [Rohingya] come to them [providers] and say ‘you cannot offer this sort of service to my wife, you have done this service without asking me’ or ‘you have convinced my wife’. (Key informant #38, Program Manager/Coordinator)

The HCPs reported having experienced how conservativeness and religion affected Rohingya women’s acceptance of MR, PAC and FP, and this was also a reason for MR with medication (MRM) being more commonly provided than MVA for MR. As expressed by one of the paramedics (#39, more than 10 years of experience), ‘the Rohingyas are conservative. They do not want to take MVA, they want to eat “dawai” (medicine) [ …] as they have to show their secret places when they take MVA I think they are completely illiterate about health.’ Some HCPs expressed that limited acceptance of services due to religion, conservativeness and a lack of awareness of SRH was the difference between providing care in a humanitarian setting and a non-humanitarian setting. These factors were also expressed as reasons for having to place more emphasis on counselling. The HCPs adjusted their counselling based on their understanding of Rohingya women’s needs and desires, using religion and motherhood in their counselling to increase acceptance of MR, PAC and FP.

We tell them it [MR] is not a sin. Because it will save your family, it will make you and your new-born child happy. You already have a child. If you have another baby now, you will get bad impact on your health. You cannot give your children enough care. So, take the MR and care for your family. (Paramedic #33, more than 10 years of experience)

The HCPs perceived the Rohingya women’s living situation in the humanitarian setting as challenging and incorporated the difficulties of having another child into their counselling to increase the acceptance of MR and FP. The HCPs also felt that the humanitarian setting affected the Rohingya women’s SRH decisions and made them vulnerable to and, at risk for violence. As one of the doctors observed (#42, 0–5 years of experience), ‘actually women are vulnerable [ …] and this is an emergency situation, you know in emergency this [rape] is very common’.

## Discussion

This study is unique as it explores the experiences and perceptions of HCPs of CAC in the humanitarian setting in Cox’s Bazar, Bangladesh as well as the barriers and facilitators in the provision of CAC.

This study finds that abortion policies at the global, national and organisational levels have both increased and decreased the access to and availability of CAC in Cox’s Bazar. While the MR policy provided a favourable legal environment at a national level, the Mexico City Policy resulted in structural and organisational challenges due to different actors’ willingness to provide the full package of CAC. The Mexico City Policy, also called the Global Gag Rule, requires foreign NGOs receiving US global health assistance to certify that they do not use the US funds or their own non-US funds to counsel clients or provide abortion services [27]. In this setting, this has resulted in discrimination against some aspects of CAC in Cox’s Bazar, which may result in unnecessary delays due to referrals and missed opportunities to meet women’s SRH needs. Further, the Mexico City Policy limits women’s and girls’ access to information on safe abortion services, as organisations and service providers that are subject to the terms of the Mexico City Policy are prohibited from providing such information [27]. The Nairobi Summit on International Conference on Population and Development + 25 (ICPD25) highlighted that women’s limited access to safe abortion remains an ‘unfinished agenda’ and called for actions to increase the provision of a comprehensive SRHR package that integrates CAC and essential parts of Universal Health Coverage (UHC) [28]. Integrating CAC services with primary health care could improve the access to and availability of CAC and a range of other health care services, as various SRH conditions are related [2].

Globally, research highlights the need to protect the SRHR of displaced and refugee populations and strengthen SRH services in humanitarian settings, including ensuring that safe abortion services are provided for every indication stated in the abortion law [2]. This requires that humanitarian actors and SRH providers have knowledge of the abortion law in the context in which they work. Inaccurate knowledge could limit or delay women’s access to abortion services, even when HCPs are willing to provide such services. Although the HCPs in this study had received training from an NGO prior to their placement in Cox’s Bazar, both doctors and paramedics had limited knowledge of the national abortion law and inconsistent knowledge of the MR policy. This knowledge gap suggests that these topics are not fully covered in the training curricula and that there is a need for continuous training, including non-clinical training. The fact that almost all of the participants had received training from NGOs means that the NGOs had an opportunity to ensure that providers are equipped with adequate knowledge to offer quality care and reduce delays due to incorrect referrals or by denying women services they are legally permitted to receive.

Unlike previous studies on abortion-related care in humanitarian settings [1, 10, 11] and in LMIC [13], our study shows that the HCPs in Cox’s Bazar felt confident and adequately trained to carry out their work, despite their lack of knowledge about the abortion law and MR policy. This stands in contrast to findings from non-humanitarian settings in Bangladesh demonstrating a lack of providers trained in MR and PAC, which limited the access to and availability of MR and PAC, even in facilities with adequate supplies and equipment [17]. The findings of this study and those of previous research in Bangladesh suggest that MR and PAC are not fully covered during medical training. This gap in training has been filled by NGOs in Cox’s Bazar. However, there is a need to institutionalise training to reduce gaps and inequalities in the access to and availability of quality MR, PAC and FP services throughout Bangladesh. Although HCPs felt adequately trained, the lack of space to ensure women’s privacy affected their ability to provide quality services in both NGO- and GoB-managed facilities.

A lack of supplies is commonly reported as a barrier in humanitarian settings [1, 11]. The reported availability of adequate supplies in Cox’s Bazar indicate a commitment of humanitarian actors as well as well-functioning coordination and supply chain management. Functional coordination and supply management was also reported by the Women’s Refugee Commission’s (WRC) case study on contraceptive services for Rohingya women and girls in Cox’s Bazar [20]. However, in this study, services were limited due to a lack of space, and implants were not available on demand since only trained doctors, who were in short supply, could provide the service. Implants can be safely and effectively administered by nurses and midwives [29], and task-shifting and task-sharing are effective strategies for increasing access to different SRH services [2]. This also applies to non-humanitarian settings in Bangladesh. An unpublished study on midwives’ experiences of SRH care in Cox’s Bazar shows that the midwives felt frustrated about not being trained to provide comprehensive SRH service and having to refer women to other providers for FP and MR [30]. This suggests that there is an opportunity to increase the provision of CAC through comprehensive training of midwives to ensure that they can confidently provide services. To support sustainability and equity in care, the training should be included in the midwifery curriculum. The midwifery cadre is fairly new in Bangladesh and thus there is room for additional research on task-sharing to provide a context-specific evaluation of the safety, effectiveness and acceptability of treatment.

Despite the availability of adequate supplies, this study reveals the presence of an informal health system in the humanitarian setting due to the availability of MR medications from untrained local pharmacists or shop keepers. This is congruent with research on MR and PAC in non-humanitarian settings in Bangladesh [17, 24]. In Cox’s Bazar, the HCPs suggested that the informal health system affected women’s trajectories of abortion care and health outcomes, reporting that incomplete abortions were a result of women taking MR medicines purchased from local pharmacies. Training informal providers and ensuring increased access to self-management of medical abortions and/or tele-medicine could help to minimise unsafe abortions. As MRM was reported to be the most common approach, there is potential to increase access to MR through telemedicine or self-medication. The presence of an informal health system indicates that CAC is not fully accessible or available in Cox’s Bazar and may suggest that the Rohingya population has limited or no access to information and knowledge about MR, PAC and FP services—or that such services are stigmatised.

Humanitarian settings can increase the vulnerability of women and girls to sexual violence and unintended pregnancies [1, 3, 4], as indicated by our study participants. The HCPs saw it as their responsibility to care for Rohingya women who were in vulnerable situations. This sense of responsibility and desire to make a difference became a motivating factor for some HCPs. Despite the accounts of the stigma associated with their work, the HCPs framed their work as something important that they took pride in. Thus, they created a positive identity, which can be a way to overcome the stigma attached to abortion work [19]. To circumvent stigma, create trust and facilitate understanding of the services provided, the HCPs adopted context-sensitive language and changed their terminology during FP and MR counselling. The use of simple language while counselling aligns with key principles of the WHO’s guideline on safe abortion and the Bangladesh MR policy [18, 22]. The strategy of changing terminology, which is not unique to this context, can ease stigmatisation and enable the provision of abortion care [19]. In this study, changing the terminology during counselling seemed to allow Rohingya women to bypass local perceptions of religious norms and the HCPs to sidestep their own religious beliefs; however, it also seemed to cement the stigma associated with the word abortion and the belief that it is a religious sin and entails ending a life.

Interpretation of religion through the dominant cultural lens and limited knowledge of SRH among Rohingya women were expressed by the HCPs as the main differences between providing CAC in the humanitarian setting in Cox’s Bazar compared to a non-humanitarian setting in Bangladesh. Rohingyas are Muslims, as are the vast majority of Bangladeshis and the HCPs. The practice of Islam and interpretation of the Quran differ across cultures, across and within nations and communities. Although some HCPs in this study sought to be culturally competent and acquired skills and applied strategies to communicate and provide care cross-culturally, their personal beliefs affected the care they provided. Some HCPs’ condescending attitudes toward Rohingya women and their tendency to view Rohingya women as a homogenous group with certain needs and desires could affect the quality of care provided. Research in a non-humanitarian setting in Bangladesh found that gendered norms and HCPs’ perceptions about clients affected the quality of care provided [23]. Making assumptions about clients’ needs and desires can take away their agency and reproductive decision-making capabilities. In this study, the HCPs’ personal beliefs and their perception of the Rohingya population led them to think it was necessary to have the husband’s consent before providing MR and FP services, and this could delay appropriate care. The MR policy in Bangladesh does not state that consent from a husband is needed to carry out an MR or that the woman’s marital status needs to be confirmed [18]. This suggests that adhering to social norms takes precedence over the policy environment, which is supported by previous research carried out in Bangladesh [17, 23, 24]. Further, the way the HCPs evoked motherhood in counselling to make MR less shameful might have been a result of their perception of the fertility preferences of the Rohingya population and/or a result of their own culture impacting their counselling practice. Evoking motherhood in counselling may produce a view that MR is acceptable only for women who have fulfilled the societal duty of becoming a mother. Previous research in Bangladesh has reported similar findings [17, 23].

To create a safe space for health care services across cultures, HCPs need to be able to analyse their own biases, prejudices and attitudes and how that affects the care they provide. Further, they need to be aware of the power relations and the power imbalance in provider–client interactions [31]. Taking a cultural safety approach to health care services in Cox’s Bazar could increase the quality of care and lead HCPs and health care organisations to examine how their culture, biases, prejudices and attitudes affect the care delivered. According to a cultural safety approach, the definition of a safe space lies with the client [31]. Taking such an approach would mean that Rohingya women are viewed as active agents who define the care they receive.

### Limitations

The interviews provided rich data and allowed us to explore a sensitive topic that is often overlooked in humanitarian settings. However, the study faced a number of limitations. Due to time and logistical constraints regarding access to the refugee camps, high workloads, distance to facilities and infrastructural challenges, it was not feasible to conduct a pilot interview. Thus, a thorough preparation meeting was held with the interpreter before the data collection started, and debriefing sessions were held after each interview to discuss the interpretation, interview flow and whether questions needed to be re-phrased. All changes were recorded. The facility set-up also posed challenges with ensuring privacy during the interviews. The interviewer (MP) ensured that the participants felt comfortable even if complete privacy could not be maintained. If the HCP did not feel comfortable, the interview would have been cancelled or postponed, but this did not happen. Even though the HCPs were open to sharing their experiences and perceptions, the fact that privacy could not be maintained at all times could have affected some of the answers. Further, the use of an interpreter could be a limitation, as an interpreter can affect a participant’s willingness to respond and can knowingly or unknowingly interpret words and phrases in ways that do not correspond accurately to those of the participant. To verify the data, one of the authors (NIP) fluent in English and Bengali, transcribed and simultaneously translated the full interview conversations with the assistance of an additional transcriber. For conceptual coherence and quality of translation, one of the authors (NIP) cross-checked the transcribed material of the other transcriber. The fact that the findings mainly represent the perspectives of paramedics is not considered to be a limitation, as most HCPs who provide MR and PAC services in the study context are paramedics.

## Conclusion

The availability and accessibility of CAC was limited by unfavourable abortion policies affecting organisations’ scope of services, which led to safe abortion services being excluded. CAC was further limited by a lack of privacy, HCPs’ lack of knowledge of the national abortion law and MR policy, HCPs’ personal beliefs and a lack of cultural safety. Advocacy for a comprehensive and holistic SRHR approach is needed to ensure that the humanitarian response, at a minimum, includes safe abortions for all indications of the law. The actors involved in delivering the SRHR response in Cox’s Bazar, can improve the access to and availability of quality CAC by working toward integrating the full package of CAC services in the health care system to address women’s multiple SRH needs, by ensuring sufficient space and privacy for CAC services and by expanding the administration of CAC to other professions, such as midwifery. Furthermore, training in cultural safety as well as in-service training, including value clarification, should be emphasised to improve the quality of care and to ensure that the care that is provided is woman-centred and non-judgmental. Additionally, the CAC curricula should include training on the abortion law and MR policy to reduce delays and ensure that women are not denied services. To fully understand the trajectories of abortion-related care in the humanitarian setting in Cox’s Bazar, more research is needed on women’s health-seeking behaviours and encounters with abortion-related care from their own perspectives.

## Supplementary Information

**Additional file 1.**

**Additional file 2.**

**Additional file 3.**

## Data Availability

The interviews and transcripts are stored at the WHO collaborating centre for Human Reproduction at Karolinska Institutet in a locked cabinet. For safety and privacy reasons, the interview materials will not be shared because they could potentially identify participants. Please contact the corresponding author with any questions.

## Abbreviations

BAPSA: Association for the Prevention of Septic Abortion, Bangladesh
CAC: Comprehensive abortion care
FP: Family planning
HCP: Health care providers
GoB: Government of Bangladesh
IDI: In-depth interviews
IOM: International Organisation for Migration
LARC: Long-acting and reversible contraceptive
LMIC: Low- and middle-income country
LMP: Last menstrual period
MR: Menstrual regulation
MRM: Menstrual regulation with medicine
MVA: Manual vacuum aspiration, an abortion method
NGO: Non-governmental organisation
PAC: Post-abortion care
PHC: Primary health centre
RH: Reproductive health
SRH: Sexual and reproductive health
SRHR: Sexual and reproductive health and rights
UN: United Nations
UNFPA: United Nations Population Fund
UNHCR: United Nations High Commissioner for Refugees
WHO: World Health Organisation
