# Supplementary material for: A qualitative study on health care providers’ experiences of providing comprehensive abortion care in Cox’s Bazar, Bangladesh
Source: Confl Health. 2021 Jan 13;15:6. doi: 10.1186/s13031-021-00338-9 (PMC7805103; doi:10.1186/s13031-021-00338-9)
Supplement: Supplementary file 1 — Additional file 1. [file 13031_2021_338_MOESM1_ESM.pdf]

Topic Guide for In-depth Interviews with Healthcare Providers

Healthcare Providers' Experience of Providing Comprehensive Abortion Care in a Humanitarian Setting

Introduction

1. **To start, could you tell me a bit about yourself?**
   - About your professional background, education and training?

2. **How did you learn how to provide MR and PAC?**
   - During education?
   - How was the quality of education/training?

Health System

3. **Could you describe your current work in Cox's Bazar?**
   - What type of services do you provide? Define concepts used: MR, FP, PAC, CAC?
   - What methods of menstrual regulations are provided? Women's acceptance of methods (medical-MVA) in general? Why?
   - What contraceptive methods do you suggest and have access to? Women's acceptance of (PAC) contraceptive counselling and methods? Why?
   - How do women know where to access the services?

4. **Could you describe how the MR/PAC/FP services are organized at your clinic?**
   - Who does what?
   - Collaboration among organizations, the government, clinics? Referral system? Outreach program?

Preparedness

5. **Could you describe what kind of training you received before starting to work in Cox's Bazar?**
   - How did your organization/employer assist in the preparations?
   - Adequately prepared to provide MR services, contraceptive counselling and PAC?

6. **How does your organization work with training of health care providers in the field?**
   - Ongoing training to maintain capacity over time?
   - Information and training on new recommended procedures and changes in policy of MR?

Experience

Interviewer: We are curious to know how about your daily work and the services you provide.

7. **Could you tell me about your last MR procedure?**
   - How did the woman find out about her pregnancy? To whom did she disclose her pregnancy? Who was involved in her decision-making?
   - How did she feel about the pregnancy?
   - What services was provided and how?
   - How would you describe the woman's acceptance of the MR method and contraceptive counselling?
   - How was your experience?

8. **If you think about your workplace, what makes it possible for you to provide MR, PAC and FP?**
   - How does your organization support you and your colleagues?
   - What are the main challenges? How does your organization deal with these challenges?
   - What makes it possible for you to provide MR services?

9. **How would you describe the difference between performing MR services in Cox's Bazar compared to your previous workplace?**
   - What are the differences in supplies, equipment, training, support, human resources?
   - How is the facility when it comes to being able to ensure privacy and confidentiality?

Socio-cultural context and knowledge environment

10. **Based on your experience here in Cox's Bazar, why do you think women have unintended pregnancies?**
    - What might be the root cause?
    - Unsafe abortions common? Why? Can you give an example?
    - Incomplete abortion? Why? Can you give an example?

Interviewer: From my experience abortion laws and policies can sometimes be difficult to interpret and understand.

11. **Could you tell me about the abortion law and MR policy in Bangladesh?**
    - How did you learn about the abort law and MR policy?
    - How would you describe the knowledge on laws and policies in society? Why does it look like this?

12. **How would you describe the difference between MR and abortion?**
    - How do you think people in your community describe the difference?

13. **How would you describe the general view on abortion?**
    - In Bangladesh?

- In the Rohingya community in Cox's Bazar?
- Difference between men and women's views?

14. How would you describe the general view on MR services?
- In Bangladesh?
- In the Rohingya community in Cox's Bazar?
- Difference between men and women's views?
- Have you ever worked with someone who did not want to perform MR services? How did that make you feel? What was your response?

15. How would you describe how are the Rohingya women seeking MR service treated by their community?
- Why do you think the community view women this way?
- What might they say? Difference between married/unmarried? Young/Adult?
- What type of support do these women have?

16. What would you say is needed in order to improve access to MR, contraceptive counselling and PAC services in Cox's Bazar?

Interviewer: Thank you very much for participating. We truly appreciate you taking time for this interview and sharing your expertise. Before we end, we wish to ask if there anything you would like to add in relation to what we have discussed or that you feel we have missed to bring up?

Socio demographic information (Collected after the IDI):

Educational background	
Occupation	
Length of working experience	
Length of working in Cox's Bazar	
Organization/ Facility setting	
