# Supplementary material for: A qualitative study on health care providers’ experiences of providing comprehensive abortion care in Cox’s Bazar, Bangladesh
Source: Confl Health. 2021 Jan 13;15:6. doi: 10.1186/s13031-021-00338-9 (PMC7805103; doi:10.1186/s13031-021-00338-9)
Supplement: Supplementary file 2 — Additional file 2. [file 13031_2021_338_MOESM2_ESM.pdf]

Topic guide for Key-Informant Interviews

Healthcare Providers' Experience of Providing Comprehensive Abortion Care in a Humanitarian Setting

Organizational environment

1. **Could you tell me a bit about the organization you work at?**
   - What is the organization's main role?
   - Could you tell me a bit about your position at the organization?

Interviewer: Organizations and institutions interact at international, regional and national and levels and shape the availability of abortion services in local contexts.

2. **How would you describe your organizations position on abortion?**
   - What is the organization's role at an international, regional, national and local level?
   - How does the organization collaborate with other organizations?
   - What do you think are the challenges/facilitators in your organization?
   - What do you think are the challenges for other organizations when it comes to their position on abortion?

3. **In the onset of a crisis how does your organization work to establish itself in the field? (in general, and Cox's Bazar)**
   - How is the organization financed?
   - How does the organization collaboration with national and local government and with other organizations in the field?
   - What guides the services provided by your organization?

4. **Could you share with me your thoughts on your organizations role when it comes to providing CAC humanitarian settings?**
   - How would you describe your organization's mandate when it comes to CAC in humanitarian settings?
   - How would you describe your organizations role in collaborating with other organizations and the government?

Health system

Interviewer: We are also interested in the structure of health care (CAC) in humanitarian settings (focus on Cox's Bazar) and how your organization position itself in relation to other organizations and how services are organized.

5. **Could you tell me about your organizations work in Cox's Bazar?**
   - Organizations role in provision of MR/PAC in Cox's Bazar?

- Collaboration with other organizations?
- How are healthcare services set up? Organizations offering same/different services? Referral systems?

6. **Could you share with me your thoughts about unintended pregnancies in Cox's Bazar?**

7. **How would you describe the general view on abortion and MR in Bangladesh/Rohingya community?**

8. **How would you describe the perception of the legality of abortion/MR in Bangladesh/Rohingya community/international humanitarian aid workers?**

9. **How does your organization work with medical training of health care providers in the field for them to be able to provide CAC?**
    - How does your organization work to maintain the capacity over time?
    - How does your organization work with evaluation of outcome of training activities?

Interviewer: Abortion laws can be difficult to understand, and abortion policies can change in response to changes in the political or religious leadership.

10. **How does your organization work with interpretation of abortion laws?**
    - Dissemination of knowledge on legality of abortion within organization and to service providers? Interpretation and the legal space? Who is legally permitted to provide abortion services?
    - How does your organization work with training on abortion legality of health care providers?

11. **How does your organization work in setting with restrictive abortion laws?**
    - How does the legal space affect the provision of CAC?
    - How does your organization work with safety of health care providers?

Socio-cultural context

12. **How does your organization work in settings where abortion is restricted by societal norms?**
    - How does your organization work with training of health care providers? Meeting women from different social context, religions etc.?
    - How does your organization work with interpretation of religion?
    - Local and national government?
    - How does your organization work with reaching women?

13. **What would you say are the challenges for your organizations when it comes to providing CAC in humanitarian settings?**
    - In what situations can your organization not provide CAC?
    - How does your organization deal with the challenges?

14. **What would you say are the enablers for your organizations when it comes to providing CAC in humanitarian settings?**
    - In what situations can your organization provide CAC?
    - How does your organization work to maintain and develop these enabling factors?

Interviewer: Thank you very much for participating. We truly appreciate you taking time for this interview and sharing your expertise. Before we end, I wish to ask if there anything you would like to add in relation to what we have discussed or that you feel I have missed to bring up?

Socio demographic information (Collected after the IDI):

Sex	
Educational background	
Professional experience (role)	
Length of working experience	
Organization	
