# Supplementary material for: A qualitative study on health care providers’ experiences of providing comprehensive abortion care in Cox’s Bazar, Bangladesh
Source: Confl Health. 2021 Jan 13;15:6. doi: 10.1186/s13031-021-00338-9 (PMC7805103; doi:10.1186/s13031-021-00338-9)
Supplement: Supplementary file 3 — Additional file 3. [file 13031_2021_338_MOESM3_ESM.pdf]

Category System

Health Care Providers' Experiences of Providing Comprehensive Abortion Care in Cox's Bazar, Bangladesh

**Open codes**	**Sub-category/**	**Category**
Good collaboration between organisations<br>Supportive colleagues<br>The referral system is disputed<br>Presence of an informal health system<br>HCP are the only provider of MR and PAC at their facility<br>HCP are not the only provider of FP – duplication of services<br>Territorial issues between organisations<br>Mexico City policy affect availability<br>Lack of space due to Mexico City Policy<br>Sufficient space<br>Strong leadership from UNFPA (SRH WG)<br>Good collaboration with GoB<br>MR policy means enabling legal environment	Organisational collaboration in a humanitarian setting	**Organisation, collaborations, and policies influencing provision of CAC**
Equipment and supplies are available<br>Implants are not available<br>IUD not available<br>More supplies in the humanitarian setting than non-hum<br>No difference between humanitarian setting and non-hum<br>Training of field facilitators - increase access<br>Lack of privacy<br>Unsafe abortion is due to taking drugs from shop<br>Incomplete abortion is due to taking drugs from shop	Availability and accessibility of MR, PAC and FP	
Feeling adequately trained<br>Feeling confident about their work<br>Lack of knowledge of abortion law<br>Knowledge and interpretation of MR policy – inconsistency<br>Gestational age for MR<br>Training received from NGO<br>Counselling is important<br>Assisting HCPs in understanding abortion law	HCPs knowledge and confidence	**Influence of confidence, competence and pride on HCPs' provision of CAC**
Feeling good providing services – saving women from danger<br>Feeling satisfied when woman accept suggested method<br>Feeling a sense of responsibility over the Rohingya women<br>Feeling bad when a woman does not accept a suggested method<br>Feeling good when woman is satisfied<br>Difficult to meet women who have been raped<br>Scared of repercussion from society and husbands	Work related emotions	

Counsel in local language<br>Language can be a barrier<br>Trust<br>Comfort<br>Establish rapport<br>Identify with women	Building trust and adopting local language	
Abortion is bad for your health<br>Abortion is PAC<br>Abortion is miscarriage<br>Abortion is after 12 weeks<br>Abortion is for victims of torture<br>Abortion is when a woman has done something<br>Abortion is a sin<br>The word abortion is not used<br>Menstrual regulation is about the menstrual cycle<br>Menstrual regulation is before the embryo is a child<br>Menstrual regulation is not terminating a pregnancy<br>Menstrual regulation is abortion<br>Menstrual regulation is not a sin	HCPs view on abortion and menstrual regulation	
Perceive permission from husband to be needed for Rohingya women<br>View fertility norms in Rohingya community as high<br>Perceive Rohingya women to be in risk of violence after MR<br>Perceive Rohingya community to not like FP<br>Perceive motherhood to be important for Rohingya women<br>Use motherhood to increase acceptance<br>View unintended pregnancy to be due to pre/extra martial relations<br>Perceive Rohingya women to have poor knowledge on health<br>Counselling husband to increase acceptance<br>Experience Rohingya women wanting to keep MR and FP a secret	HCPs perception about sexual and reproductive health and decision-making in the Rohingya community	**Influence of HCPs' understanding of Rohingya women's needs on CAC provision**
Perceive Rohingya women to be helpless<br>Perceive crowded houses to be reasons for GBV and rape<br>Perceive crowded houses to be reason for unintended pregnancy<br>Experience that uncertainty of future impacts service acceptance<br>Perceive alternative costs to impact Rohingya women's behaviour<br>Acceptance of MR and FP through counselling on economic benefits	HCPs perception about Rohingya women's life in statelessness	
Perceive Rohingya women to be very religious<br>Perceive Rohingya women to be conservative<br>Think abortion is a sin for Rohingya women<br>Think Rohingya women don't want to expose themselves<br>Perceive Rohingya women to be scared of services<br>Perceive FP to be a sin in the Rohingya community<br>Think MVA is a sin in the Rohingya community<br>Think Rohingya women are scared of going to hell<br>Using religion to increase acceptance of MR	HCPs perception about religion and in the Rohingya community	

Pre-marital sex is not ok<br>Unmarried women are illegal clients<br>Unmarried women need to maintain rules to get service<br>Check marital status before MR<br>No difference between married and unmarried<br>HCP want husband's permission before providing MR and FP	HCPs personal values and attitudes about women's sexuality	
